# Combined preoperative concentrations of CEA, CA 19-9, and 72-4 for predicting outcomes in patients with gastric cancer after curative resection

**DOI:** 10.18632/oncotarget.9060

**Published:** 2016-04-27

**Authors:** Xuechao Liu, Haibo Qiu, Jianjun Liu, Shangxiang Chen, Dazhi Xu, Wei Li, Youqing Zhan, Yuanfang Li, Yingbo Chen, Zhiwei Zhou, Xiaowei Sun

**Affiliations:** ^1^ Sun Yat-Sen University Cancer Center, State Key Laboratory of Oncology in South China, Collaborative Innovation Center for Cancer Medicine, Guangzhou 510060, China; ^2^ Department of Gastric and Pancreatic Surgery, Sun Yat-Sen University Cancer Center, Guangzhou, China

**Keywords:** CTM, combination of preoperative tumor markers, prognosis, scoring system, tumor markers

## Abstract

In many cancers, prognostic factors are useful for identifying high-risk patients and in individualizing treatment. We sought to determine whether a combination of tumor markers (CTM) would improve prognostic accuracy in patients with gastric cancer (GC). The CTM score, which is derived from serum concentrations of carcinoembryonic antigen (CEA), carbohydrate antigen 19-9 (CA 19-9), and carbohydrate antigen 72-4 (CA 72-4), was tested retrospectively in 1134 patients with GC undergoing curative resection between October 2000 and December 2012. The CTM score was 2 for patients with two or three elevated markers, 1 for those with one elevated marker, and 0 for those no elevated markers. Overall survival (OS) in patients with CTM scores 0, 1, and 2 was 61.8%, 31.4%, and 15.1%, respectively (P<.001). The CTM score independently predicted OS on multivariate analysis (HR, 1.95; 95% CI, 1.73 to 2.21; *P*<.001). Moreover, the area under the receiver operating characteristics curve of the CTM score (0.67; 95% CI, 0.64 to 0.70) was higher than the values of any individual marker (0.63, 0.57, 0.57; P<.001 for all comparisons). The CTM score independently predicted postoperative survival in GC, and it may have better clinical utility than individual tumor markers for identifying high-risk patients with GC.

## INTRODUCTION

Gastric cancer (GC) is one of the most common and deadly malignancies worldwide, with a high incidence of recurrence and metastasis, even after radical surgery [1, 2]. Despite advances in surgery and multidisciplinary treatment, the long-term postoperative survival of patients with advanced-stage GC still remains low [3, 4].

In many cancers, independent prognostic factors have been useful in identifying high-risk patients and in adjusting treatment. The tumor-nodes-metastasis (TNM) system has been the reference standard for assessing GC prognosis. However, the prognosis for patients with GC can vary, even when they have the same TNM stage, and the most accurate TNM staging requires waiting for postoperative histologic results [5]. Therefore, clinicians and researchers continue to seek other prognostic factors that might help improve the clinical management of patients with GC.

In a variety of cancers, tumor markers have been useful in assessing prognosis and tailoring treatments [6–8]. These markers, which are secreted either by the tumor or as a response to the tumor, have also been widely used for cancer screening, diagnosis, and postsurgical surveillance[9, 10]. The most common markers used in GC have been carcinoembryonic antigen (CEA), carbohydrate antigen 19-9 (CA 19-9), and carbohydrate antigen 72-4 (CA 72-4) [11–13]. The value of using these individual tumor markers in GC for prognostic assessment has been questioned because of their low sensitivity and high false-positive rate [14]. However, recent reviews have suggested that, when combined, these three markers performed better than when used alone, both in staging before chemotherapy and surgery and in improving sensitivity without impairing specificity [14, 15].

Given these results, we postulated that a specific combination of preoperative serum tumor markers might be useful in determining the prognosis in patients with GC. We developed a new combination scoring system involving CEA, CA 19-9, and CA 72-4, which we named the CTM (combination of preoperative tumor markers) score, and validated the score with a retrospective study to determine whether the CTM score was an independent prognostic factor for GC. Our goal was to determine the CTM scores of our patients with GC and to compare these scores to a variety of clinicopathological variables, including overall survival (OS).

## RESULTS

Of the 1134 enrolled patients (770 men), mean (range) age at the time of diagnosis was 56.6 (18 to 86) years; 232 patients were stage I, 290 were stage II, and 612 were stage III (Table 1) [5]. OS after a median follow-up of 36 months (range 1 − 162) was 65.6%, so 744 patients were alive at last follow-up. Tumor markers CEA, CA 19-9, and CA 72-4 were elevated in 22.1%, 18.0%, and 21.0% of patients, respectively.

**Table 1:** General characteristics of 1134 gastric cancer patients associated with overall survival

	No. of patients(%)	OS (months) mean(95% CI)	P-value^a^
Age (<60 / ≥60 years)	650 (57.3%) / 484 (42.7%)	103.8 (96.5, 111.1) / 76.5 (67.6, 85.4)	<0.001
Sex (Male / Female)	770 (67.9%) / 364 (32.1%)	91.6 (84.6, 98.6) / 95.5 (85.6, 105.4)	0.987
Tumor location	254 (22.4%) / 223 (19.7%)/	65.2 (57.0, 73.4) / 60.5 (48.3, 72.6) /	<0.001
(Upper / Middle / Lower third)	657 (57.9%)	105.9 (98.5, 113.3)	
Tumor size	129 (11.4%) / 450 (39.7%)/	130.9 (120.0, 141.7) / 98.5 (90.2, 106.7) /	<0.001
(<3 / 3≤ diameter <5 / ≥5)	555 (48.9%)	76.2 (68.1, 84.2)	
Histological grade	218 (19.2%) / 916 (80.8%)	100.9 (90.3, 111.5) / 89.3 (82.5, 96.1)	0.009
(Well / Poorly differentiated)			
Depth of invasion	220 (19.4%) / 92 (8.1%)/	122.9 (112.5, 133.2) / 117.8 (100.7, 135.0) /	<0.001
(T1 / T2 / T3 / T4)	277 (24.4%) / 545 (48.1%)	86.0 (75.0, 97.0) / 64.4 (59.1, 69.7)	
Nodal status	370 (32.6%) / 176 (15.5%)/	122.7 (112.7, 132.7) / 106.5 (94.8, 118.2) /	<0.001
(N0 / N1 / N2 / N3)	207 (18.3%) / 381 (33.6%)	83.8 (71.7, 95.9) / 56.0 (48.2, 63.9)	<0.001
TNM stage (I / II / III)	232 (20.5%) / 290 (25.6%)/	141.2 (131.6, 150.8) / 106.0 (94.9, 117.2) /	<0.001
	612 (54.0%)	68.9 (61.8, 76.1)	
CEA (Normal / Elevated)	883 (77.9%) / 251 (22.1%)	106.0 (99.5, 112.5) / 54.6 (46.1, 63.1)	<0.001
CA72-4 (Normal / Elevated)	896 (79.0%) / 238 (21.0%)	101.2 (95.0, 107.5) / 64.6 (53.4, 75.8)	<0.001
CA19-9 (Normal / Elevated)	930 (82.0%) / 204 (18.0%)	99.7 (93.4, 106.1) / 56.9 (48.1, 65.6)	<0.001
CTM (0 / 1 / 2)	632 (55.7%) / 340 (30.0%)/	115.4 (108.1, 122.7) / 74.1 (63.8, 84.4)/	<0.001
	162 (14.3%)	52.0 (41.8, 62.2)	

Abbreviations: OS = overall survival; CI = confidence interval; TNM = tumor-node-metastasis staging; CEA = carcinoembryonic antigen; CA = carbohydrate antigen; CTM = the combination of preoperative tumor markers;

a Kaplan–Meier survival analysis.

**Figure 1:**
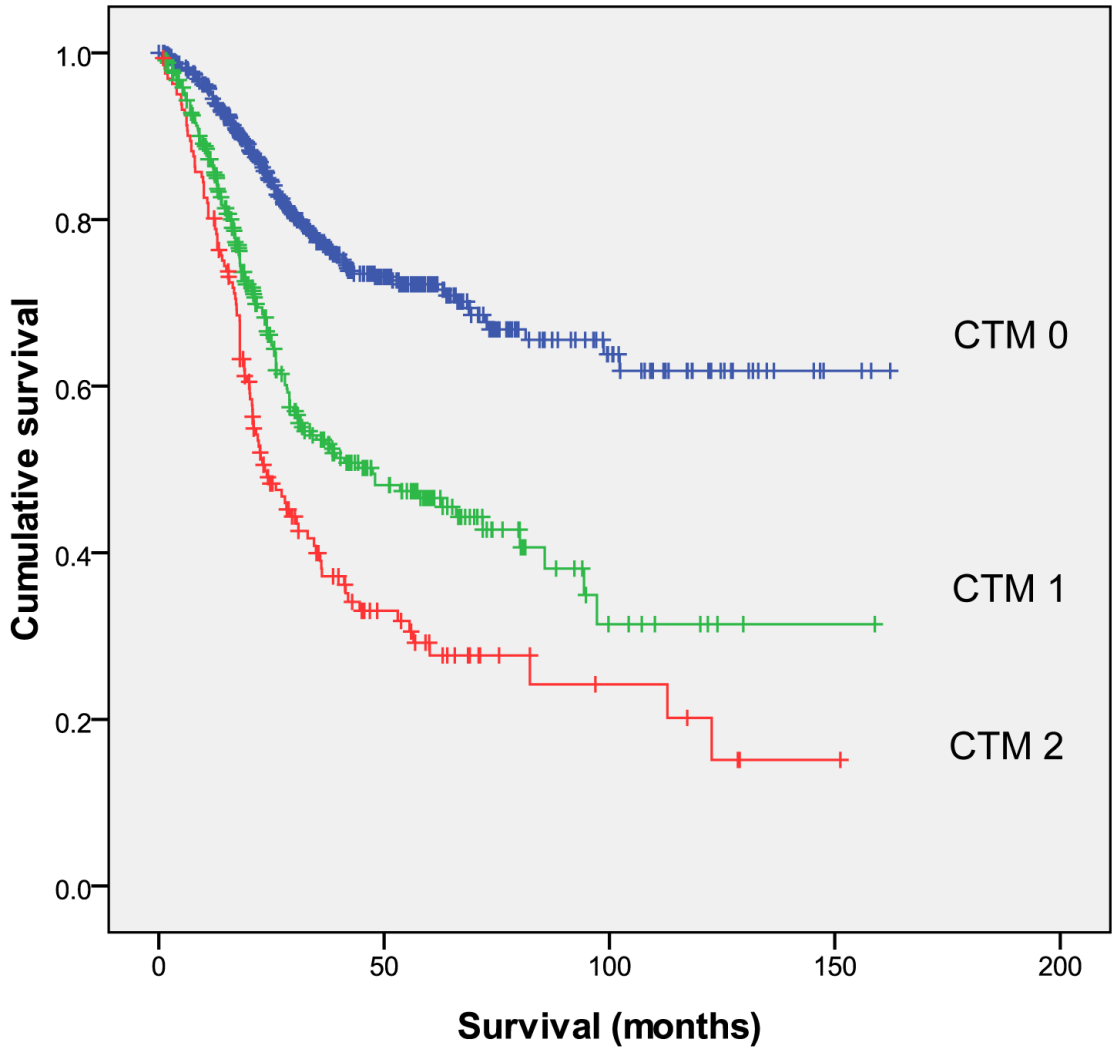
Relationships among the three CTM groups (CTM 0, CTM 1 and CTM 2, from top to bottom) and overall survival CTM = the combination of preoperative tumor markers.

We divided patients into three independent groups by CTM score (CTM 0, no elevated markers, n=632; CTM 1, one elevated marker, n=340; and CTM 2, two or three elevated markers, n=162), and evaluated the association of each group with OS (Figure 1). We used a CTM of 2 for either two or three elevated markers because we found that there was no statistically significant difference in OS between patients with two and patients with three elevated markers (P = 0.001) (Supplementary Figure S1). The sensitivity and specificity for the CTM were 64.1 and 66.1 %, respectively. However, we found that, the sensitivity was rather low for combination of any two tumor markers. The sensitivity and false positive rate were 53.8 and 26.7 %, 55.4 and 22.6 %, and 45.4 and 26.3 % for combination of CEA and CA 72-4, CEA and CA 19-9, and CA 72-4 and CA 19-9, respectively.

The OS rates in patients with CTM scores 0, 1, and 2 were 61.8%, 31.4%, and 15.1%, respectively (P <.001). Patients with a CTM score of 0 lived significantly longer (median, 115.4 months) than did patients with a CTM score of 1 or 2 (P <.001; Table 1). Furthermore, when patients were stratified by TNM stages I through III, the CTM score was still associated with OS within each TNM stage (P=.001, P=.004, and P<.001, respectively).

Univariate analysis showed that 11 clinicopathological variables had significant univariate associations with OS (Table 2). When we used the absolute concentrations of CEA, CA19-9, and CA72-4 instead (data not shown), their prognostic value were rather limited and only the concentration of CA72-4 was independently associated with OS in univariate analysis (HR, 1.004; P<.001). After excluding correlated variables, six remaining variables were entered into the multivariate analysis. A CTM score of 2 (HR, 1.51), TNM stage 4 (HR, 2.41), tumor location in the lower third of the stomach (HR, 0.74), and age (HR, 1.68) were independently associated with OS (Table 2). The multivariate analysis also showed that CEA (HR, 1.71) and CA 19-9 (HR, 1.28) were independently associated with OS, whereas CA 72-4 (HR, 1.25; P=.051) was only marginally.

**Table 2:** Univariate and multivariate analyses of overall survival in 1134 gastric cancer patients

	Univariate analysis		Multivariate analysis	
	HR (95 % CI)	P-value	HR (95 % CI)	P-value
Age (<60 / ≥60 years)	1.694 (1.388, 2.067)	<0.001	1.675 (1.369, 2.050)	<0.001
Sex (Male / Female)	1.002 (0.809, 1.241)	0.987		
Tumor location	0.686 (0.614, 0.766)	<0.001	0.742 (0.663, 0.831)	<0.001
(Upper / Middle / Lower third)				
Tumor size	1.821 (1.540, 2.154)	<0.001	1.090 (0.908, 1.309)	0.356
(<3 / 3≤ diameter <5 / ≥5)				
Histological grade	1.442 (1.095, 1.899)	0.009	1.219 (0.921, 1.613)	0.167
(Well / Poorly differentiated)				
Depth of invasion	1.574 (1.413, 1.754)	<0.001		
(T1 / T2 / T3 / T4)				
Nodal status	1.801 (1.641, 1.975)	<0.001		
(N0 / N1 / N2 / N3)				
TNM stage (I / II / III)	2.813 (2.345, 3.375)	<0.001	2.405 (1.965, 2.944)	<0.001
CEA (Normal / Elevated)	2.683 (2.188, 3.290)	<0.001		
CA72-4 (Normal / Elevated)	1.893 (1.524, 2.352)	<0.001		
CA19-9 (Normal / Elevated)	2.046 (1.635, 2.561)	<0.001		
CTM (0 / 1 / 2)	1.953 (1.728, 2.207)	<0.001	1.505 (1.323, 1.712)	<0.001

Abbreviations: HR = hazard ratio; CI = confidence interval; TNM = tumor-node-metastasis staging; CEA = carcinoembryonic antigen; CA = carbohydrate antigen; CTM = the combination of preoperative tumor markers.

Higher CTM scores were associated with older age (≥ 60 years), larger tumor size (≥ 5 cm), tumor location in the upper third of the stomach, and higher TNM stage (Table 3). However, CTM scores were not associated with sex or histological grade.

**Table 3:** Correlation between CTM and clinicopathologic factors

	CTM 0	CTM 1	CTM 2	P value
	(n = 632)	(n = 340)	(n = 162)	
Age (<60 / ≥60 years)	391 / 241	187 / 153	72 / 90	<0.001
Sex (Male / Female)	426 / 206	233 / 107	111 / 51	0.923
Tumor location	120 / 111 / 401	86 / 73 / 181	48 / 39 / 75	<0.001
(Upper / Middle / Lower third)				
Tumor size	92 / 275 / 265	31 / 129 / 180	6 / 46 / 110	<0.001
(<3 / 3≤ diameter <5 / ≥5)				
Histological grade	118 / 514	70 / 270	30 / 132	0.747
(Well / Poorly differentiated)				
Depth of invasion	168 / 58 / 136 /270	44 / 28 / 90 / 178	8 / 6 / 51 / 97	<0.001
(T1 / T2 / T3 / T4)				
Nodal status	270 / 108 / 91 / 163	81 / 48 / 82 / 129	19 / 20 / 34 / 89	<0.001
(N0 / N1 / N2 / N3)				
TNM stage (I / II / III)	181 / 185 / 266	46 / 76 / 218	5 / 29 / 128	<0.001

Abbreviations: TNM = tumor-node-metastasis staging; CTM = the combination of preoperative tumor markers.

**Figure 2:**
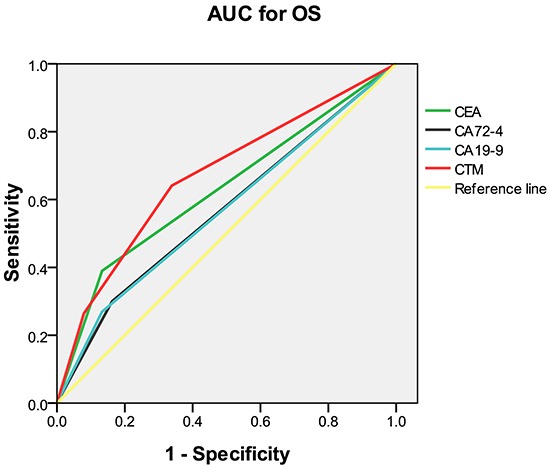
Comparison of the areas under the curves for survival prediction AUC = area under the curve; OS = overall survival; CEA = carcinoembryonic antigen; CA = carbohydrate antigen; CTM = the combination of preoperative tumor markers.

The discriminatory ability of elevations in individual and combined tumor markers was assessed with receiver operating characteristic (ROC) curves by comparing areas under-the-curve (AUC) (Figure 2). The CTM score had a higher AUC value (0.67; P<.001) than any of the individual tumor markers (CEA, 0.63; CA 19-9, 0.57; CA 72-4, 0.57; Table 4).

**Table 4:** Comparison of the areas under the curves for overall survival

	AUC	95% CI	P-value
CEA	0.628	(0.593, 0.664)	<0.001
CA72-4	0.569	(0.533, 0.605)	<0.001
CA19-9	0.568	(0.532, 0.604)	<0.001
CTM	0.670	(0.637, 0.704)	<0.001

Abbreviations: AUC = area under the curve; CI = confidence interval; CEA = carcinoembryonic antigen; CA = carbohydrate antigen; CTM = the combination of preoperative tumor markers.

## DISCUSSION

In our study of patients with GC, we used a combination of three tumor markers, CEA, CA 19-9, and CA 72-4, to develop a new scoring system, which we called CTM (combination of preoperative tumor markers). Our goal was to determine the prognostic value of CTM scores in patients with GC undergoing curative resection. We found that the CTM score was independently associated with OS in GC and this association held up in GC TNM stages I, II, and III.

The most common tumor markers used in patients with GC have been CEA, CA 19-9, and CA 72-4, but none of these markers are currently recommended for use in the screening, diagnosis, or postsurgical surveillance of GC by the current National Comprehensive Cancer Network Clinical Practice Guidelines. The potential value of these tumor markers was addressed by a recent meta-analysis of 33 studies. This showed that CEA, CA19-9, and CA 72-4 performed better in combination than individually, by improving sensitivity without impairing specificity, when used for screening, diagnosis and follow-up monitoring of GC [15].

We were interested in evaluating these tumor markers as prognostic factors that might help improve the clinical management of patients with GC. Although some studies evaluating the prognostic value of these markers have reported mixed results, other studies have suggested that elevated CEA, CA 19-9, and CA 72-4 concentrations may be associated with tumor progression and may provide additional prognostic information in GC [12, 16–20].

We noticed in clinical practice that patients with GC who had more than one elevated tumor marker tended to have poorer outcomes. With this in mind, we postulated that a combination of tumor markers might provide more meaningful prognostic information than individual tumor markers. We created a combined tumor marker scoring system (CTM), and we compared the CTM scores of our patients to their OS. In our system, a CTM score of 0 was given to those patients with no elevated markers, and a score of 1 was given to those with only one elevated marker. Because there was no significant difference in OS in patients with two and patients with three elevated markers, we merged these and gave them a CTM score of 2. Our work has some precedent, in that a similar but more complex, combined tumor marker scoring system has been used by others for GC [13]. However, we believe the CTM scoring system is a less complicated system which would be more practical and efficient to implement in the clinical setting.

We found that CTM was independently associated with OS. We also determined that CEA and CA 19-9 were independently associated with OS, and that CA 72-4 was marginally associated with OS. However, the AUC value was higher for CTM than that for any of the individual markers, suggesting that CTM was a better discriminatory variable for OS.

These results are supported by other studies. One group found that elevated serum concentrations of CEA, CA 19-9, and CA 72-4 were significantly associated with lower 3-year cumulative survival rates in patients with GC [21]. Another study reported that CEA and CA 19-9 concentrations were independent prognostic factors for GC [11]. Finally, others have reported that although individual tumor markers were not independently associated with survival, elevations in all preoperative CEA, CA 19-9, and CA 72-4 concentrations were associated with a worse prognosis in patients with GC. The authors concluded that when used together, the combination of these three markers might provide additional prognostic information in patients having surgery for GC and that patients with a preoperative elevation of any of these three biomarkers should be considered to be at high risk for recurrence, even in early GC [22].

Why the combination of elevated preoperative tumor marker concentrations was associated with reduced OS remains unclear [23]. The mechanisms of action of these tumor markers may provide some insight into why their elevation is associated with a poorer prognosis. CEA is important in promoting tumor cell adhesion and signal transduction. Moreover, given that CEA and proliferating cell nuclear antigen are closely related, CEA may also be involved in tumor cell proliferation [24]. Likewise, CA 19-9 is important in the adhesion of tumor cells to endothelial cells, so cells expressing this substance may have greater invasive and metastatic potential [25]. Carbohydrate antigen 72-4 has also been associated with both tumor cell adhesion and tumor metastasis [8, 26]. The specific activities of these substances may explain why their high serum concentrations are associated with a poor prognosis.

The CTM score was associated with OS within each TNM stage, suggesting that the CTM score has prognostic value in GC stages I, II, and III. Thus, the CTM score may help clinicians identify high-risk patients earlier, particularly those thought to be at low-risk because of a low TNM stage. Indeed, patients with a low TNM stage but a high CTM score may benefit from closer monitoring or more aggressive adjuvant therapy. We agree with others that it might be worth exploring the use of GC tumor markers and CTM score in the early postoperative period to help guide clinical decision-making [27].

We also found that the CTM score was associated with tumor size, depth of tumor invasion, and lymph node status. These relationships suggest that higher CTM scores are more likely to be found in patients with higher tumor burdens. However, we and others speculate that another cause of higher CTM scores could be the presence of micrometastases [28]. The presence of occult metastases could certainly explain why some apparent low-risk patients as indicated by TNM stage have higher CTM scores and poor outcomes.

Our study has several limitations. It was a single-center rather than multicenter investigation. Nevertheless, one benefit of this arrangement was that surgical procedures (R0 resection plus D2 lymphadenectomy), laboratory assays, and follow-up evaluations were standardized and consistent during the study period. In addition, our low- and high-risk patients had different postoperative treatment (adjuvant chemotherapy), which may have confounded the results. Finally, we used OS as our primary outcome, and our study may have been strengthened by the use of other survival measures, such as disease-free survival. As a result, our conclusions might need to be validated with the use of additional outcome measures.

In conclusion, CTM is independently associated with OS in GC, suggesting that it can be considered a valuable independent prognostic marker in patients undergoing curative resection for GC. The CTM score may have better clinical utility than individual tumor markers for identifying high-risk patients with GC.

## MATERIALS AND METHODS

The study was approved by the Research Ethics Committee at the Cancer Center of Sun Yat-sen University. All patients provided written informed consent before we performed our retrospective analysis of their medical records.

### Study population

We retrospectively analyzed clinicopathological data from 1134 patients with GC who underwent surgical resection at Sun Yat-sen University Cancer Center between October 2000 and December 2012. All patients had histologically confirmed stage I through III gastric adenocarcinoma, as defined by the 7th edition of the American Joint Committee on Cancer (AJCC) tumor-nodes-metastasis (TNM) staging system [5].

Experienced surgeons performed total gastrectomy with D2 lymphadenectomy following the Japanese Research Society for Gastric Cancer (JRSGC) guidelines[29]. All patients had gastric resections in which margins were microscopically negative and no gross or microscopic tumor remained in the primary tumor bed (R0). Patients with stage III or high-risk stage II GC and no marked comorbidities precluding chemotherapy had their cases discussed at a multidisciplinary conference and were offered 5-fluorouracil-based (5-FU) adjuvant chemotherapy after surgery. Patients who met all of the following criteria were included in the study: 1) complete clinicopathological and follow-up data, 2) no neoadjuvant chemotherapy or radiotherapy, 3) no other synchronous malignancy, 4) no evidence of distant metastases, and 5) no residual or recurrent GC.

The clinicopathological characteristics of all patients were abstracted from our hospital information system. The following variables were collected for each patient: age, sex, preoperative laboratory measurements, surgical pathology report, and time between surgery and the last available follow-up visit or death, whichever came first. Patients with papillary and well- or moderately-differentiated GC were included in the well-differentiated histology group, and those with undifferentiated, signet ring cell, and mucinous GC were included in the poorly-differentiated histology group [30].

### Follow-up

Patients were followed clinically every 3 months during the first 2 years after surgery and every 6 months thereafter until at least 5 years after surgery or until they died, whichever came first. The final follow-up date for the study was July 2015. Postoperative follow-up assessment involved medical history, physical examination, laboratory testing, upper gastrointestinal endoscopy, and dynamic chest/abdominal computerized tomography (CT) scan.

### Tumor markers

Preoperative CEA, CA 19-9, and CA 72-4 concentrations were measured within 2 weeks of surgery by enzyme immunoassay. Based on the manufacturer's instructions, the cutoff thresholds for elevated concentrations of CEA, CA 19-9, and CA 72-4 were 5 ng/mL, 27 U/mL, and 5 U/mL, respectively. Patients with two or three elevated markers received a CTM score of 2, those with one elevated marker received a score of 1, and those with no elevated marker received a score of 0.

### Statistical methods

Results are presented as means and 95% confidence intervals. Differences among the groups were analyzed using the Pearson chi-square test and Kruskal-Wallis test. Survival analysis was performed with the Kaplan-Meier method, and differences between survival curves were compared with a log-rank test. Variables significant at the 0.05 level in the univariate or unadjusted analysis were selected for inclusion in a final multivariate Cox proportional hazards model. Variables were assessed for interaction and co-linearity.

## SUPPLEMENTARY FIGURE


