# Supplementary material for: Combined preoperative concentrations of CEA, CA 19-9, and 72-4 for predicting outcomes in patients with gastric cancer after curative resection
Source: Oncotarget. 2016 Apr 27;7(23):35446–53. doi: 10.18632/oncotarget.9060 (PMC5085242; doi:10.18632/oncotarget.9060)
Supplement: Supplementary file 1 [file oncotarget-07-35446-s001.pdf]

SUPPLEMENTARY FIGURE

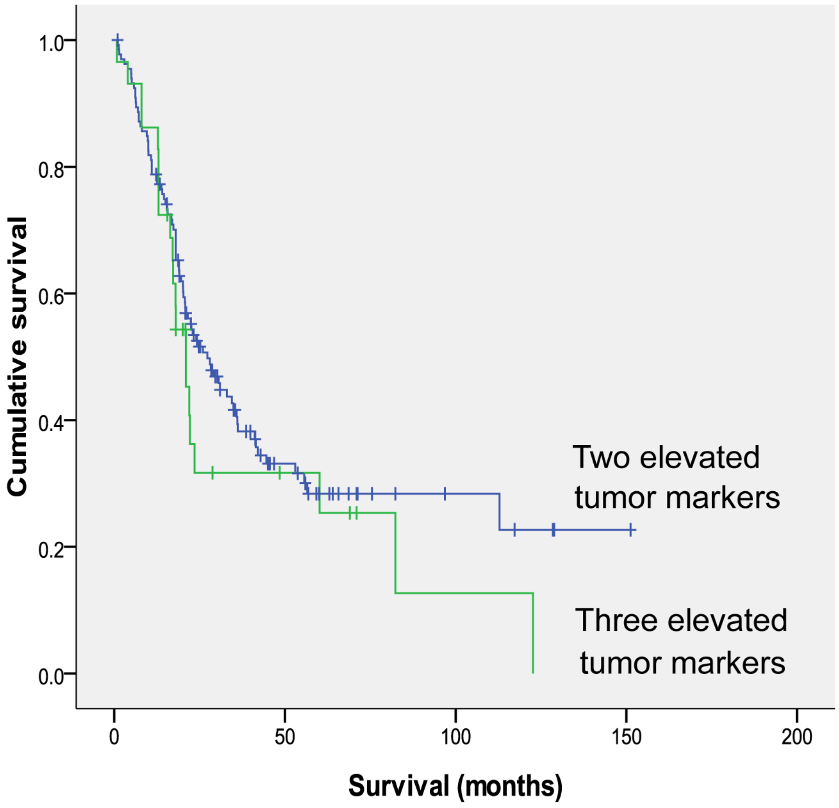

Supplementary Figure S1: Overall survival based on the number of elevated tumor markers (2 and 3, from top to bottom).
